# The emerging clinical relevance of genomic profiling in neuroendocrine tumours

**DOI:** 10.1186/s12885-021-07961-y

**Published:** 2021-03-06

**Authors:** Guney Isa Burak, Sonmezler Ozge, Mujde Cem, Buyukdereli Gulgun, Dogruca Yapar Zeynep, Bisgin Atil

**Affiliations:** 1 Cukurova University Faculty of Medicine Nuclear Medicine Department of Balcali Hospital and Clinics, Adana, Turkey; 2 Cukurova University AGENTEM (Adana Genetic Diseases Diagnosis and Treatment Center), Adana, Turkey; 3 Cukurova University AGENTEM & Cukurova University Faculty of Medicine Medical Genetics Department of Balcali Hospital and Clinics, Adana, Turkey

**Keywords:** Neuroendocrine tumours, Next generation sequencing, Genomic profiling, Somatic mutation, Liquid biopsy, Circulating cell-free DNA

## Abstract

**Background:**

Neuroendocrine tumours (NETs) arise from hormone-producing or nervous system cells and can develop from anywhere in the body. They have heterogeneous origins from skin to gastrointestinal track and a complicated histology. Thus, there is an inevitable need for genomic profiling to determine the exact genetics of each tumour for prognosis and treatment strategies to overcome the disease’s complexity. For this purpose, next-generation-sequencing (NGS) is the most reliable methodology for both germ-line and somatic studies to make a clinical diagnosis. In this study, we analyse liquid biopsies, formalin fixed paraffin embedded (FFPE) tissues, and peripheral blood samples for their ability to provide information for actionability.

**Methods:**

A customized multi-gene panel comprised of Succinate Dehydrogenase Complex Iron Sulfur Subunit B (*SDHB),* Succinate Dehydrogenase Complex Subunit C *(SDHC),* Cell Division Cycle 73*(CDC73),* Calcium Sensing Receptor *(CASR),* Platelet Derived Growth Factor Receptor Alpha *(PDGFRA),* Succinate Dehydrogenase Complex Flavoprotein Subunit A *(SDHA),* Ret Proto-Oncogene *(RET), Succinate Dehydrogenase Complex Assembly Factor 2(SDHAF2),* Menin 1*(MEN1),* Succinate Dehydrogenase Complex Subunit D *(SDHD),* MYC Associated Factor X *(MAX)* and Protein Kinase CAMP-Dependent Type I Regulatory Subunit Alpha *(PRKAR1A)* genes was constructed to assess multiple specimen types including: 3 liquid biopsies, 6 FFPE tissues, and 26 peripheral blood samples from 35 unique NET patients. Quality-control and bioinformatics analyses were performed using QCI-Analyze and QCI-Interpret.

**Results:**

The three liquid biopsies and the 6 FFPE tissue samples were evaluated for somatic mutations; while the 26 peripheral blood samples were analysed using the germ-line pipeline. Five (55.6%) of the nine patients that were studied for somatic changes carried actionable mutations related to therapy sensitivities. Through the germ-line studies, we observed a 50% positivity rate for disease predisposition with 16 variants classified according to ACMG (American College of Medical Genetics) Standards and Guidelines.

**Conclusions:**

Genomic profiling medicine is an emerging area of clinical oncology and has become crucial for disease and patient management by providing a precision approach; this is especially true for rare diseases including rare cancers such as NETs. Notably, this study emphasized the relevance of multiple distinctive biological sample types for use in the genetic testing of cancers to help with the choice of therapy to maximize the likelihood of a positive clinical outcome.

## Background

Neuroendocrine tumours (NETs) are a relatively rare group of cancers which can arise from endocrine and nervous system cells. They can originate from multiple tissues and organs; most frequently from within the lungs, pancreas and intestine. Although the gastroenteropancreatic neuroendocrine tumours group (GEP-NET) is the major class which includes pancreatic neuroendocrine tumours and carcinoids, thymus, parathyroid and lung forms are also take place in rarer cases [1]. The definition and classification of NETs has changed over time due to their complexity [2, 3]. The International Agency for Research on Cancer (IARC) and the World Health Organization (WHO) have published a site-specific criteria and terminology guide to provide uniform classifications This organ-specific approach has aided more accurate nomenclature and grading. Moreover, it has provided us with a better understanding of NETs’ organ-specific characteristics and prognostic behaviours [4, 5]. Due to the fact that NETs can develop anywhere in the body and that the organ-tissue of origin plays an important role in prognosis and response to treatments, the patients’ and the tumour’s genetic backgrounds have become more important. The conventional treatment strategies included mainly surgery. However, it is now known that a multidisciplinary approach is necessary for a more personalized and efficient therapy taking into consideration of site of origin, potential of metastasis, grade and the genetic background of the patient. Finally, the heterogeneous origins of NETs lead to a very complicated tumour histology and multiple distinctive aetiologies [6, 7].

Although the vast majority of NETs are sporadic and caused by somatic mutations, germ-line genetic changes also play a critical role in disease inheritance and prognosis [8, 9]. Therefore, a patient-specific approach is a necessity for disease management [7, 10]. Next-generation sequencing (NGS) has become the most powerful methodology for the precise diagnosis, prognosis track and development of therapeutic strategies for many types of cancers. Through the development and testing of customized gene panels for various cancer types, it has been possible to provide precision medicine. In our centre we perform NGS from liquid biopsies - one of the most popular topics in oncology - on formalin fixed paraffin embedded (FFPE) tissues, and peripheral blood samples for the accurate genomic profiling of NETs to determine their hereditary predispositions and treatment actionability.

## Methods

### Demographic and clinical information

Thirty-five distinctive NET patients who were referred to our center for molecular genetic testing were recruited for this study. Patient distribution by their tumor site of origin is as follows; 74.3% (*n* = 26) foregut (including lung, pancreas, thymus, parathyroid, stomach and medullary thyroid cancer), 5.7% (*n* = 2) hindgut (rectum cancer), 17.1% (*n* = 6) other (given in Table 1) and 2.9% both foregut (pancreas cancer) and hindgut (rectum cancer). All participants signed an informed consent, and were enrolled in accordance with the ethical standards of the institutional ethical committee (Cukurova University Faculty of Medicine Non-Invasive Clinical Research Ethics Commission) and the Helsinki declaration. Starting material types were selected in accordance with the patients’ diagnoses, clinical background and familial history. Patients with any inherited syndromes suspected were excluded from the study. Demographic information for the patients is shown in Table 1.

**Table 1:** Demographic information of the patients, biological sample types, sites of origin and World Health Organization (WHO) classification of NETs

Patient	Material	Site of Origin	WHO Classification
P1	Liquid Biopsy	Foregut (Lung)	G1
P2	Liquid Biopsy	Foregut (Pancreas)	G2
P3	Liquid Biopsy	Foregut (Pancreas)	G3
P4	FFPE tissue	Foregut (Thymus)	G1
P5	FFPE tissue	Other (Surrenal cortical carcinoma, NET)	G3
P6	FFPE tissue	Hindgut (Rectum)	G2
P7	FFPE tissue	Foregut (Thyroid)	G1
P8	FFPE tissue	Foregut (Pancreas)	G3
P9	FFPE tissue	Other (Urinary system, NEC)	G3
P10	Peripheral Blood	Foregut (Thyroid)	G1
P11	Peripheral Blood	Foregut (Parathyroid)	G1
P12	Peripheral Blood	Foregut (Thyroid)	G1
P13	Peripheral Blood	Foregut (Pancreas)	G3
P14	Peripheral Blood	Foregut (Stomach)	G2
P15	Peripheral Blood	Other (Primary location unknown)	G3
P16	Peripheral Blood	Foregut (Thyroid)	G1
P17	Peripheral Blood	Foregut (Thyroid)	G1
P18	Peripheral Blood	Foregut (Thyroid)	G2
P19	Peripheral Blood	Foregut (Thyroid)	G1
P20	Peripheral Blood	Hindgut (Rectum)	G3
P21	Peripheral Blood	Foregut (Thyroid)	G1
P22	Peripheral Blood	Foregut (Thyroid)	G2
P23	Peripheral Blood	Foregut (Pancreas)	G3
P24	Peripheral Blood	Foregut (Pancreas)	G2
P25	Peripheral Blood	Foregut (Pancreas)	G1
P26	Peripheral Blood	Foregut (Pancreas)	G1
P27	Peripheral Blood	Foregut (Pancreas) + Hindgut (Rectum)	G3
P28	Peripheral Blood	Foregut (Pancreas)	G3
P29	Peripheral Blood	Foregut (Pancreas)	G1
P30	Peripheral Blood	Other (Primary location unknown, Hepatic and Pancreas NET)	G3
P31	Peripheral Blood	Foregut (Pancreas)	G1
P32	Peripheral Blood	Foregut (Pancreas)	G3
P33	Peripheral Blood	Foregut (Pancreas)	G1
P34	Peripheral Blood	Other (Head and Neck NET)	G3
P35	Peripheral Blood	Other (Paraganglioma, NET)	G2

*G* Grade, *NET* Neuroendocrine Tumour, *NEC* Neuroendocrine Carcinoma. Age range in males: 17–76. Age range in females: 15–77

### Sampling and DNA isolation

Liquid biopsy samples were collected via biological sampling tubes special for ccfDNA. Tumor sites were determined to obtain FFPE tissue sections. Somatic DNA from liquid biopsies (circulating cell-free DNA, ccfDNA) and FFPE tissues were isolated by manufacturer’s instructions with modifications [QIAamp Circulating Nucleic Acid Kit and DNA FFPE Tissue Kit (Qiagen, Germany)] [11, 12]. Genomic DNA from peripheral blood was isolated via instructions provided by the kit manufacturer [QIAamp DNA Blood Midi Kit (Qiagen, Germany)]. Fluorometric measurements were made using a Qubit 3.0 fluorometer to assess the quality and quantity of the isolated DNAs. The purified DNAs were then subjected to next-generation sequencing.

### Next-generation sequencing and QC

Target enrichment was performed starting with 40 ng of input gDNA from PBMCs, 100 ng of DNA from peripheral blood ccfDNA from liquid biopsies; and 250 ng of DNA from FFPE samples using a customized-designed multi-gene capture panel (Qiagen, Hilden, Germany) that consists of 12 NET-related genes. All exons and exon-intron junctions of Succinate Dehydrogenase Complex Iron Sulfur Subunit B *(SDHB),* Succinate Dehydrogenase Complex Subunit C *(SDHC),* Cell Division Cycle 73 *(CDC73),* Calcium Sensing Receptor *(CASR),* Platelet Derived Growth Factor Receptor Alpha *(PDGFRA),* Succinate Dehydrogenase Complex Flavoprotein Subunit A *(SDHA),* Ret Proto-Oncogene *(RET),* Succinate Dehydrogenase Complex Assembly Factor 2 *(SDHAF2),* Menin 1 *(MEN1),* Succinate Dehydrogenase Complex Subunit D *(SDHD),* MYC Associated Factor X *(MAX)* and Protein Kinase CAMP-Dependent Type I Regulatory Subunit Alpha *(PRKAR1A)* genes were amplified and sequenced with a minimum of 300x coverage for germ-line studies and 1500x coverage for somatic studies.

Sequencing quality control assessments were carried out using the QCI-Analyze tool, and the QCI-Interpret interface was used for the bioinformatics analyses. Multiple quality control parameters including total yield, variant frequency, forward/reverse ratio, depth of coverage and quality score were assessed for each sample type. All of the detected genetic changes were evaluated independently of the patients’ diagnoses. Next generation sequencing steps and quality control assessments were performed with optimization based on previous studies [11, 12].

### Data interpretation and bioinformatics analysis

Bioinformatics analyses were made for each variant detected using multiple databases including: HGMD (Human Gene Mutation Database), ClinVar, NCBI (National Center for Biotechnology Information), VarSome (The Human Genomic Variant Search Engine), ExAC (The Exome Aggregation Consortium), 1000 Genome Frequency, ESP (Exome Sequencing Project), Ancestry, Ingenuity Knowledge Base, COSMIC, GnomAD, OMIM (Online Mendelian Inheritance in Man) and Franklin to provide as much information as possible for making each clinical interpretation. All of the *in-silico* predictions were performed using a minimum of 10 different analysis tools including MutationTaster, SIFT and PolyPhen 2. The pathogenicity classification of each of the detected variants was determined in compliance with ACMG guidelines and standards.

In the secondary analysis, all of the variants which met the quality control criteria were investigated in accordance with the patients’ diagnoses and clinical findings. Hereditary predispositions were determined for germ-line studies, and actionability was assessed for the detected somatic variants.

## Results

Total of thirty-five patients were recruited to the study which 25.7% (*n* = 9) of them are for somatic and 74.3% (*n* = 26) of them are for germline molecular testing. Six patients of 9 somatic studies were analysed for somatic variants in FFPE samples with their NET diagnoses. Actionable mutations with regard to therapy were detected in 3 (50%) of them. Patients had six actionable mutations in *PDGFRA*, *RET* and *SDHA* genes. Among the liquid biopsy studies, we detected four actionable mutations within the *RET* oncogene in two (75%) of the three liquid biopsy specimens. Mutations were observed in the same clone for one of these patients while the other patient had the mutation in different clones. The distribution of the detected somatic alterations is given in Table 2. The overall positivity rate was 55.6% among the somatic studies. Additionally, somatic status of *PDGFR* variant with 49% allel frequency was confirmed by the re-evaluation with germ-line study.

**Table 2:** Distribution of the actionable variants which all are in confidentiality rates according to the literature and our previous clinical validation studies [11–13]

	Patient	Anatomic location	Gene	Variant (amino acid change)	Allel frequency (%)
Liquid Biopsy	P2	Pancreas	*RET*	D698fs*71	0.65
				N950fs*9	0.54
	P3	Pancreas	*RET*	N950fs*9	1.63
				L19del	1.48
FFPE tissue	P4	Other	*PDGFRA*	T463S	49
	P5	Other	*RET*	L390G	0.94
			*SDHA*	G112fs*49	0.72
	P9	Other	*SDHA*	K541*	5.8
				R465Q	14
				S456L	28

*F* Female, *M* Male

* symbol were used to indicate stop codon formation as recommended in HUGO Gene Nomenclature Committee standards

Through the germ-line studies, we also observed a 50% positivity rate for disease predisposition with 16 variants identified among 13 patients. Germ-line mutations were seen in the *RET*, *KIT*, *SDHB*, *SDHC*, *SDHD* and *PDGFRA* genes. The variant distributions and their classification criteria are listed in Table 3 according to the ACMG Standards and Guidelines.

**Table 3:** Implemented variant classification criteria of detected germ-line mutations

Patient	Gene	Variant (amino acid change)	ACMG criteria (doi: 10.1038/gim.2015.30)
P11	*KIT*	M541L (Heterozygote)	PM2, BP4, BP6
	*SDHD*	G12S (Heterozygote)	PP2, BS1, BS3, BP4, BP6
P14	*SDHB*	G19FS*57 (Heterozygote)	PVS1, PM1, PM2
P15	*PDGFRA*	S66R (Heterozygote)	PM2, BP1, BP4
P17	*RET*	G691S (Heterozygote)	PP2, BA1, BS3, BP4
P18	*RET*	G691S (Heterozygote)	PP2, BA1, BS3, BP4
P19	*RET*	G691S (Heterozygote)	PP2, BA1, BS3, BP4
P22	*RET*	G691S (Heterozygote)	PP2, BA1, BS3, BP4
	*KIT*	A431E (Heterozygote)	PM1, PM2, BP4
P24	*RET*	G691S (Homozygote)	PP2, BA1, BS3, BP4
	*RET*	A1051V (Heterozygote)	PM1, PP2, PP3
P25	*RET*	G691S (Heterozygote)	PP2, BA1, BS3, BP4
P29	*KIT*	R946* (Heterozygote)	PVS1, PP3, BS2
P30	*RET*	A96V (Heterozygote)	PM2, PP2, BP4
P34	*KIT*	M541L (Heterozygote)	PM2, BP4, BP6
P35	*SDHC*	R72H (Heterozygote)	PM1, PM2, PP3

*PVS1* Pathogenic Very Strong, *PP2* *and* *PP3* Pathogenic Supporting, *PM1 and PM2* Pathogenic Moderate, *BP1, BP4 and BP6* Benign Supporting, *BA1and BS3* Benign Strong

* symbol were used to indicate stop codon formation as recommended in HUGO Gene Nomenclature Committee standards

**Fig. 1:**
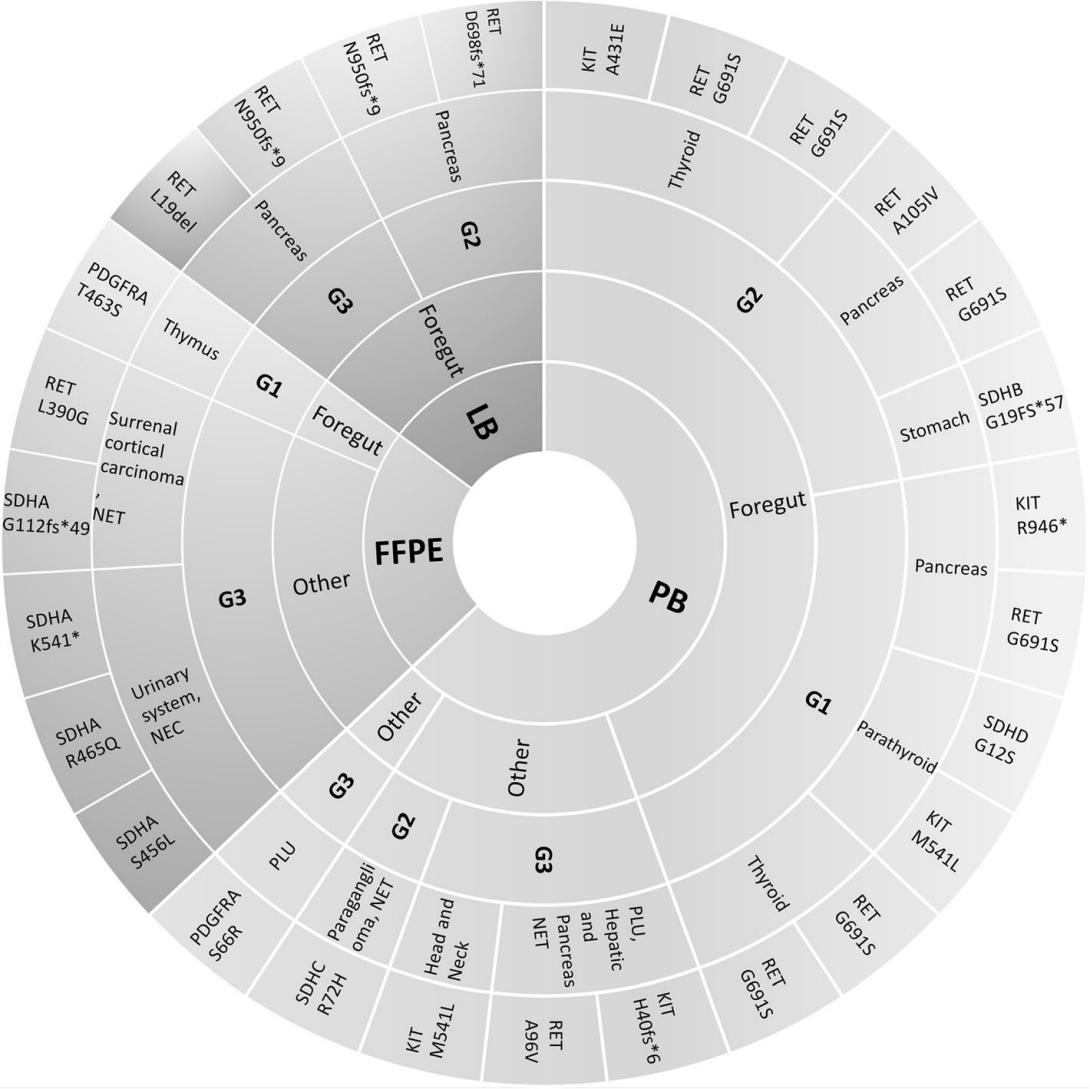
Variant distribution versus clinical characteristics. PB: Peripheral Blood, LB: Liquid Biopsy, FFPE: Formalin Fixed Paraffin Embedded Tissue, G: Grade, PLU: Primary Location Unknown, NET: Neuroendocrine Tumour, NEC: Neuroendocrine Carcinoma

Additionally, variation quantiles according to grade and site of origin were given in Fig. 1. The overall mutation distribution was not distinctive between neither the different material types nor the severity of diseases.

This study evaluated both the risk factors and proposed potential risk factors together with germline and somatic genomic profiling. A multi-gene panel was used to investigate the genetics underlying both germline and somatic NETs and was demonstrated to provide actionable information in greater than 50% of the cases including those were evaluated using liquid biopsy specimens.

## Discussion

Comprehensive molecular genetic profiling is a must for heterogeneous group of cancers such as NETs. As a result, we detected somatic variations in *RET, PDGFRA* and *SDHA* genes; while *RET, KIT, SDHB, SDHC, SDHD* and *PDGFRA* genes were mutated in germ-line studies. The distribution of the detected variants and genes are deviational because of the complex tissue origins of NETs and the individual differences. When the genetic changes classified by the ACMG criteria and assessed together with the both biological sample type and pathological findings, the frequency becomes similar between the groups. A comprehensive grade specific study with a broaden cohort is needed due to more precise variant interpretation and molecular grade classification of NETs.

We also emphasized that not only the somatic changes play a role in cancer progression and therapeutic approach, but a complement study of germ-line genetic profiling is beneficial for the patient. Cote et al. has shown that the liquid biopsy plays a prognostic role on medullary thyroid carcinoma due to a single-variant study of *RET* oncogene, and can be efficiently used in monitoring the disease [14]. Cavalcanti et al. recently reported that tracking *PDGFRA* expressions provide as a promising anti-angiogenic target in well-differentiated NETs [15]. Knösel et al. also showed that the over-expression of *KIT* and *PDGFRA* genes are related to short survival and a negative prognostic factor in advanced pancreatic neuroendocrine tumours [16]. Niemeijer et al. reported that SDH mutations also cause other types of cancers which have neuroendocrine origins other than paraganglionic tumours. Literature of NETs points that a broaden genetic profiling of both somatic and germline mutations should be the next step to improve NET diagnostics and therapeutic approaches. Emerging the accurate material type with the proper multi-gene panels that next generation sequencing technologies offer is the key.

## Conclusions

Molecular genetic testing strategies can be tailored to fit an individual patient’s specific needs. The most efficient approach including the best specimen type and target gene panel will differ for each patient group, particularly those that are rare and/or heterogeneous cancers such as NETs. Genomic profiling medicine can act as a bridge between clinicians and patients for to provide precision for the development of therapeutic algorithms. Additionally, it is also crucial to practice effective patient follow-up to ensure proper patient management for both the patients and their families.

In summation, in this study we show the significance of selecting determinative biological samples in the decision making process to provide the best possible health care service.

## Data Availability

The datasets used and/or analysed during the current study are available from the corresponding author upon reasonable request.

## Abbreviations

NET: Neuroendocrine Tumours
NGS: Next Generation Sequencing
GEP-NET: Gastroenteropancreatic Neuroendocrine Tumours
DNA: Deoxyribonucleic Acid
ccfDNA: Circulating cell-free DNA
gDNA: Genomic DNA
FFPE: Formalin Fixed Paraffin Embedded
ACMG: American College of Medical Genetics and Genomics
SDHB: Succinate Dehydrogenase Complex Iron Sulfur Subunit B
SDHC: Succinate Dehydrogenase Complex Subunit C
CDC73: Cell Division Cycle 73
CASR: Calcium Sensing Receptor
PDGFRA: Platelet Derived Growth Factor Receptor Alpha
SDHA: Succinate Dehydrogenase Complex Flavoprotein Subunit A
RET: Ret Proto-Oncogene
SDHAF2: Succinate Dehydrogenase Complex Assembly Factor 2
MEN1: Menin 1
SDHD: Succinate Dehydrogenase Complex Subunit D
MAX: MYC Associated Factor X
PRKAR1A: Protein Kinase CAMP-Dependent Type I Regulatory Subunit Alpha
HGMD: Human Gene Mutation Database
NCBI: National Center for Biotechnology Information)
ExAC: The Exome Aggregation Consortium
ESP: Exome Sequencing Project
COSMIC: Catalogue of Somatic Mutations
OMIM: Online Mendelian Inheritance in Man
ACMG: American College of Medical Genetics
IARC: International Agency for Research on Cancer
WHO: World Health Organization
